# Do disadvantaged schools have poorer teachers? Rethinking assumptions about the relationship between teaching quality and school-level advantage

**DOI:** 10.1007/s13384-021-00460-w

**Published:** 2021-07-28

**Authors:** Jennifer Gore, Felicia Jaremus, Andrew Miller

**Affiliations:** Teachers and Teaching Research Centre, School of Education, The University of Newcastle, University Drive, Callaghan, NSW 2308 Australia

**Keywords:** Quality Teaching Rounds, Quality teaching, Professional development, Equity

## Abstract

Improving educational performance, including narrowing equity gaps, is frequently touted as a matter of improving the quality of teachers in the lowest performing, often disadvantaged, schools. However, the assumption that teaching is of poorer quality in disadvantaged schools is largely unsubstantiated. Using the Quality Teaching Model of pedagogy, we observed 832 lessons in 193 New South Wales primary schools and found a small relationship between teaching quality and school-level advantage. However, when 174 teachers from across the school spectrum participated in Quality Teaching Rounds we found equivalent, and substantial, gains in teaching quality across all levels of school advantage. This result indicates that differences in teaching quality are less a reflection of teacher capabilities than of the challenging circumstances in disadvantaged schools. We argue that policies seeking more equitable achievement should address wider social inequities, rather than unfairly blaming teachers for being unable to level an unequal playing field.

## Introduction

The educational opportunities, experiences, and outcomes of Australian school students have become increasingly stratified since the late 1980s. This stratification has been propelled by inequitable models of school funding and a proliferation of school choice policies fuelled by a neoliberal education agenda (Bonnor & Shepherd, 2017; Kenway, 2013; Perry, 2018). As a consequence, Australia now has one of the largest private education sectors in the world (Dinham, 2015; Perry et al., 2016), and invests well above the Organisation for Economic Co-operation and Development (OECD) average in the private education sector (OECD, 2020). Such investment contributes to the perpetuation of one of the most highly segregated education systems in the OECD (Bonnor & Shepherd, 2017; OECD, 2018) and to stark resourcing differences between schools and school sectors (Bonnor & Shepherd, 2017; Rowe & Perry, 2020). Over the past few decades, these inequities have manifested alongside growing performance gaps between the most and least advantaged schools, as well as a decline in Australia’s overall international testing results (Kenway, 2013; OECD, 2018). At the age of 15, students from low socioeconomic status (SES) backgrounds achieve, on average, three schooling years behind their more advantaged peers, as measured by the Programme for International Student Assessment (PISA) exam (OECD, 2018). In short, arguably, it is not entirely unfair to conclude that Australia provides students with “neither high quality nor high equity education, and certainly not both” (Kenway, 2013, p. 288).

During the past two decades, Australian educational policy has focussed more on marketisation, standardisation, and homogenisation of teaching, curriculum, and assessment than addressing growing resource gaps and school socioeconomic segregation (Gorur, 2013; Vickers, 2015). Following the lead of many western nations including the United States (US) and the United Kingdom (UK), Australia has facilitated neoliberal encroachment into the education landscape by positioning schools, and particularly teachers and teaching, as in need of fixing (Dinham, 2015). Teaching in Australia is repeatedly positioned by political leaders and the media as being in ‘crisis’ (Baroutsis & Lingard, 2017; Burnett & Lampert, 2016; Dinham, 2015; Shine, 2015). Teacher education is similarly labelled as ineffective (Graham et al., 2020), graduating poor quality teachers (Gorur, 2013), and filled with too many ‘poor aptitude’ students (Leigh & Ryan, 2008). Reviews, reports, and investigations into the teaching profession have concurrently ballooned (Burnett & Lampert, 2016; Rowan et al., 2015).

This broader turn against the teaching profession is particularly marked in so-called underperforming schools (Gorur, 2013). The common argument made is that improving Australia’s educational performance is a matter of improving the quality of teachers in the lowest performing, generally disadvantaged, schools (Baroutsis & Lingard, 2017; Dinham, 2013; Gannicott, 2016; Gorur, 2013; Rice, 2010). Publicly available metrics for comparing ‘like’ schools, particularly the Index of Community Socio-Educational Advantage (ICSEA),^1^ have been especially influential in supporting this argument. As Gorur (2013) notes, when one school underperforms compared to a school ‘like’ it, the ‘remedy’ proposed by government leaders is to raise the quality of teaching.

In recent years, governments and education departments have responded to the proposed teaching ‘crisis’ by attempting to change the composition of the teaching workforce. These efforts have included increased regulation and surveillance of pre-service teachers; the introduction of new academic benchmarks for those wishing to enter teacher education degrees; as well as literacy and numeracy tests for pre-service teachers (Fitzgerald & Knipe, 2016). Recently, the public school system in New South Wales (NSW) announced that teachers entering the system will also be required to demonstrate ‘superior’ cognitive and emotional intelligence, through measures such as high university achievement (Turner & Stough, 2020). The beliefs underpinning such gatekeeping mechanisms seem to be that (1) the teaching profession has a supply problem, with some of those who entered teaching in the past unfit to do so; (2) under school market conditions these teachers primarily end up in disadvantaged schools, as evidenced by the relative ‘underperformance’ of those schools; and (3) the practice of ‘poor quality’ teachers cannot be improved, hence the need to prevent certain people from entering not only the profession but also teacher education programmes.

However, as many scholars have pointed out (Burnett & Lampert, 2016; Dinham, 2015; Thrupp, 2007; Vickers, 2015), there is limited empirical basis for these assumptions or the related claims about a teaching ‘crisis’. Despite teachers being viewed as both a major problem and a solution to Australia’s growing social inequalities in recent years, few studies have actually examined the quality of teaching across advantaged and disadvantaged school contexts. Furthermore, evidence from our own studies demonstrates that teaching practice can indeed be improved (Gore et al., 2017), with teacher capability unlikely to be the ‘issue’. Indeed, evidence that teachers, while the most important measured in-school contributor to student achievement, may have less influence on achievement than students’ background characteristics (Borman & Kimball, 2005; Huang, 2015) is often neglected in policy discussions about teachers and school performance (Vickers, 2015).

In this paper, we provide a long overdue contribution to this teaching quality debate by investigating the relationship between school-level advantage and teaching quality and whether teaching quality can be improved across the socioeconomic spectrum of schools. In so doing, we question whether there is, in fact, a teaching quality ‘crisis’ (Dinham, 2015) and if calls for higher quality teaching and teachers in disadvantaged schools are empirically justifiable.

## Defining and measuring quality teaching

Conclusions about whether students in disadvantaged schools experience poorer quality teaching depend on how teaching quality is defined and measured. As a discipline, we are yet to agree upon what ‘good’ teaching looks like or, in fact, on how, and if it is even possible, to measure it (Gore, 2021; Barnes & Cross, 2018; Desimone & Long, 2010). Classroom teaching is undeniably complex and measuring a practice so intricate is challenging. Not surprisingly, then, with no internationally accepted measure of teaching quality, it is measured diversely, and often poorly (Coe et al., 2014; Rowan et al., 2015). The extant measures can be divided into four general categories: teacher characteristics; value-added models (VAMs); student ratings; and classroom observations (Coe et al., 2014; Ingvarson & Rowe, 2008; Scholes et al., 2017). Each category evaluates different phenomena as a proxy for teaching quality, has its own limitations, and tells us something different about the relationship between school-level advantage and teaching quality.

### Teacher characteristics

Teacher characteristics consist of the “personal traits, skills, and understandings an individual brings to teaching, including dispositions to behave in certain ways” (Darling-Hammond et al., 2012, p. 200). These measures, including prior academic achievement, personality, experience level, and qualification level, have been the main target of recent teacher education policies in Australia (e.g. Fitzgerald & Knipe, 2016; Turner & Stough, 2020). However, such measures are relatively static and weak proxies for teaching; they provide *no* direct information about classroom practice (Hill et al., 2015; Ingvarson & Rowe, 2008). Indeed, the relationship between teacher characteristics and student outcomes is typically mixed, small, or non-existent (Burroughs et al., 2019; Rockoff et al., 2011), suggesting that these measures often tell us little about what teachers *do*.

However, when teacher characteristics are used as a proxy for teaching quality, clear differences in quality emerge between more and less advantaged Australian schools. For example, research shows that disadvantaged schools struggle to attract adequate numbers of staff (OECD, 2016), particularly in rural areas (Sullivan et al., 2013), are less likely to attract the highest performing graduates (Burnett & Lampert, 2016), and are more likely to have staff teaching out-of-field (McKenzie et al., 2014). Disadvantaged schools also tend to have slightly younger staff (McKenzie et al., 2014), and greater numbers of inexperienced teachers (Johnston & Hayes, 2007; Luschei & Jeong, 2018; McKenzie et al., 2014) than advantaged schools. While these trends are certainly worthy of attention, it is unclear whether they tell us anything about *teaching* in different contexts. In fact, evidence from England, where less experienced teachers are also more likely to work in disadvantaged schools, suggests this pattern may reflect the preference of early career teachers to ‘make a difference’ (Allen & Sims, 2018); thus, these teachers may be the most dedicated to these contexts. The 2013 *Staff in Australia’s schools* survey supports this interpretation, where teachers in medium and low SES schools report greater commitment to the teaching profession through intending to remain working in schools for more years on average than those in high SES schools (McKenzie et al., 2014).

### Value-added models (VAMs)

VAMs seek to identify what teachers ‘add’ to students’ standardised test scores over time, usually a school year. In directly examining student outcomes, VAMs are internationally regarded as one potential solution to identifying good teachers and teaching. However, VAMs only measure student growth in a narrow part of the curriculum and face potentially irresolvable problems with reliability, validity, and bias (Hallinger et al., 2014). While VAMs are sophisticated statistical models that account for many variables which influence student achievement, they are subject to overstating the influence of teachers on student outcomes due to their inability to account for everything that influences student achievement (Hallinger et al., 2014). There are many ways, for example, that student-background and school-environment characteristics can be controlled for. When these contextual controls are not ‘aggressive’ enough, VAMs systematically assign higher rankings to teachers in advantaged schools (Ehlert et al., 2014). Furthermore, VAMs are sensitive to class composition, yielding inconsistent results for individual teachers over time (Darling-Hammond et al., 2012). Therefore, while VAMS give insight into factors that promote student achievement growth, they have been labelled by courts in America as largely ‘unfair’ measures of teacher performance (Amrein-Beardsley & Close, 2019), particularly when used in high-stakes situations and on their own.

By contrast to teacher quality measures, VAMs rarely feature as a measure of quality in Australian studies. The nature of Australia’s standardised testing system, with students tested every two years instead of every year, has hindered such developments on a wide scale (Leigh, 2010; Rice, 2010). In the U.S., these models show that teachers in disadvantaged schools tend to teach students who demonstrate less growth on standardised tests (Goldhaber et al., 2015, 2018), with some exceptions (Mansfield, 2015). However, any claims that this evidence means teachers are ‘less effective’ in disadvantaged schools (Goldhaber et al., 2015) do rely on contextual sensitivity being sufficiently addressed and the questionable assumption that raising student scores in disadvantaged settings is no more challenging than raising student test scores in more advantaged contexts (Ingersoll, 2003). On a positive note, Goldhaber et al. (2007) found that teachers in the U.S. who raise their students’ test scores the most are more likely to remain in disadvantaged schooling contexts.

### Student evaluations

Student ratings are a relatively cost-effective way to gather information about teachers and, in particular, their teaching (van der Scheer et al., 2019). Students can be asked a wide range of questions about classroom practice and multiple responders are available for a single classroom teacher (Coe et al., 2014). While this offers an opportunity to measure the quality of teaching, as experienced by students, the reliability and validity of student ratings has been questioned. Some scholars suggest student ratings are popularity contests (Fauth et al., 2014), while others claim that students do not always know what helps them learn. For example, Kornell and Hausman (2016) demonstrate that, in higher education, challenging content can lead to lower student evaluations, even when it leads to greater learning outcomes. Furthermore, student evaluations show biases for teacher gender and race (Macnell et al., 2015), adding an additional layer of unfairness.

The few studies that use student evaluations across socio-educational schooling contexts in Australia show mixed results. Using a large and nationally representative dataset, Perry et al. (2016) found few substantive differences between advantaged and disadvantaged contexts. In particular, “teachers’ use of structuring and scaffolding strategies, one of the main measures of effective teaching, varies very little across school contexts” (Perry et al., 2016, p. 186). Such evidence suggests that teaching quality across socio-educational contexts may be more consistent than commonly assumed. However, Marks (2017) challenges Perry et al. (2016), highlighting considerable differences between school sectors, with non-government schools hosting ‘superior’ learning environments according to their students. More recently, Thomson (2020) has also highlighted that fewer disadvantaged than advantaged Australian students report receiving support from their English teachers in most or every class. Together these studies indicate that there are likely to be some differences between contexts, but the nuances of these differences are poorly understood.

### Classroom observations

Classroom observations are considered by many scholars to provide an ‘unobstructed view’ of what it is that teachers *do* (Coe et al., 2014; Gitomer, 2019; Martinez et al., 2016). Of the four measures, classroom observations come closest to measuring *teaching* in a reliable and valid way. Reflecting these strengths, observations are usually given greater or equal weight in teacher evaluation internationally with measurements such as VAM scores (Goldhaber, 2015). However, observations vary depending on the measurement framework used and are the most expensive to conduct (van der Scheer et al., 2019). Currently, many different frameworks compete (Coe et al., 2014; Martinez et al., 2016) and there is no consensus on what should be included in such a framework. Furthermore, accumulating evidence indicates that context and observer biases may influence a teacher’s evaluation score (Milanowski, 2017). Nevertheless, Coe et al. (2014) suggest that classroom observations, when conducted by specifically trained observers, offer levels of reliability for measuring teaching quality that are acceptable for low-stakes purposes.

To date, few Australian studies focused on SES have measured teaching quality using observations. An earlier report from our team measured teaching quality across school contexts using the Quality Teaching (QT) Model—a model of pedagogy long endorsed by the NSW Department of Education (NSW Department of Education and Training [NSW DET], 2006)—and found a ‘miniscule’ relationship between teaching quality and school-level advantage (Gore et al., 2016). An additional study from our team has demonstrated that teaching quality, as measured by the QT Model, can be improved through a professional development (PD) program called Quality Teaching Rounds (QTR) (Gore et al., 2017). However, this study did not examine whether the school context influences improvement, an important consideration for this study. It is clear that more research is needed before evidenced-based conclusions can be drawn about whether students in disadvantaged schools experience poorer quality teaching.

## How quality teaching is defined in this study

Challenges in defining, let alone measuring, teaching quality clearly provide significant barriers to investigating whether teaching quality really is poorer in disadvantaged Australian schools. Current measures, each with their own strengths and limitations, provide different perspectives with which to view and answer the question of how school advantage relates to teaching quality. These circumstances raise important questions about how to measure teaching quality.

In this study, we build upon our prior research on the QT Model and QTR form of PD by using observational evaluation to measure teaching quality across contexts. This body of work has utilised, and continues to utilise, observational evaluation for a multitude of reasons, including (1) a core assumption of our research team is that all teachers, particularly when given the right support and opportunities for development, can improve their practice, thus we focus on measuring teaching not characteristics of teachers; (2) teachers and school leaders find observational evaluation to be fair because it assesses practices that teachers themselves can observe and work to improve (Cohen & Goldhaber, 2016; Goldring et al., 2015); and (3) at present, observations provide the most ‘unobstructed’ view of classroom practice possible (Martinez et al., 2016), enabling us to directly comment on what is actually happening in classrooms.

### The Quality Teaching Model

Derived from a long academic lineage, including work on Authentic Pedagogy (Newmann et al., 1996) and Productive Pedagogy (Ladwig, 2007), the QT Model is a comprehensive pedagogical model that can be used to assess teaching quality. It is typically applied to analysis of whole lessons, as has been done for all our research studies to date. The QT Model has already been widely implemented in Australia and has been found to not only transform teaching practice across subjects and student year levels (Gore & Rosser, 2020; Gore et al., 2017) but, crucially, to improve student outcomes when used in combination with QTR (Gore et al., 2021). Thus, while there is no consensus on what should be included in an observation framework, the QT Model is one that drives meaningful impact for teachers and students.

As shown in Table 1, the QT Model incorporates three dimensions of pedagogy: Intellectual Quality; Quality Learning Environment; and Significance. Each dimension includes six elements, which collectively honour the complexity of teaching and resonate with teachers. Further information can be found in NSW DET (2006, 2020) and Gore (2007).

**Table 1:** The Quality Teaching Model

Intellectual Quality	Quality Learning Environment	Significance
Deep Knowledge	Explicit Quality Criteria	Background Knowledge
Deep Understanding	Engagement	Cultural Knowledge
Problematic Knowledge	High Expectations	Knowledge Integration
Higher-order Thinking	Social Support	Inclusivity
Metalanguage	Students’ Self-regulation	Connectedness
Substantive Communication	Student Direction	Narrative

### Quality Teaching Rounds

QTR is a teacher PD program that embeds the QT Model and involves four (or more) teachers conducting a set of teaching ‘Rounds’ in a professional learning community (PLC), using the QT Model to discuss each other’s teaching.

This PD approach is highly suited to considering teacher capabilities across contexts because all school-based rounds are managed by the teachers themselves, with no external facilitation or oversight. Prior to the commencement of the Rounds, two teachers (usually per PLC or per school) attend a two-day (10-h) in-service workshop. This means that any change in QT scores observed post intervention can be attributed to the combination of the workshop (which is the same for teachers in all contexts) and, critically, the work that teachers undertake themselves in their own schools.

Each Round is conducted over a single day and involves the following:

1. *Reading discussion:* Professional reading chosen by one PLC member, employed to build a sense of professional community and support the development of a shared theoretical basis (approximately 1 h).
2. *Observation:* One PLC member teaches a full-length lesson that is observed by all PLC members (approximately 30–80 min).
3. *Coding and discussion:* All PLC members, including the observed teacher, individually code the observed lesson using the QT Model. Each element is coded on a scale from one to five using detailed descriptors (see NSW DET, 2020). An extended discussion follows in which PLC members discuss the lesson and teaching in general.

A set of Rounds is complete when every PLC member has been observed.

## The Study

In this paper, we investigate the popular, yet largely unsubstantiated, assumption that teaching quality is poorer in disadvantaged schools within the Australian context. Our research questions (RQs) are as follows:

1. What is the relationship between school-level advantage and teaching quality?
2. To what extent can teaching quality be enhanced in different socioeconomic school contexts through QTR?

To answer these questions, we draw on data collected as part of studies conducted in 2014–2015 and in 2019–2020. Both studies were randomised controlled trials conducted in New South Wales (NSW) government schools. The detailed design of the studies is available in the published protocols (Gore et al., 2015; Miller et al., 2019). University and state school ethics approvals were obtained for both RCTs prior to any data collection.

Three cohorts of NSW government primary school teachers are drawn from these studies; one cohort from Study 1 and two cohorts from Study 2. In brief, Cohort 1 involved primary school teachers across Stages 2–3 (Years 3–6) and includes two QTR intervention groups (a “set” group that undertook the intervention as prescribed by the research team and a “choice” group who were able to adapt the intervention by, for example, having more than four teachers in their Professional Learning Community (PLC) or conducting more than one ‘Round’ per teacher) as well as a control group. All teachers were observed by blinded members of the research team who were trained to conduct observations before QTR commenced, six months later when the two intervention groups had completed the intervention, and again six months after that to assess longer term efficacy. Approximately eight teachers were observed for each intervention school, with two whole lessons observed at each time point for most participants.

Cohort 2 involved Stage 2 (Year 3 and 4) teachers and includes two QTR intervention groups (a group whose workshops were led by the researchers and a group whose workshops were led by teachers trained by the research team), as well as an alternative intervention group and a control group. Teachers in this cohort were observed by blinded, trained members of the research team before the intervention and again eight months later when the QTR intervention was completed. Typically, two teachers were observed from each school involved in this cohort and two whole lessons were observed at each time point for each teacher where possible.

Cohort 3 also involved Stage 2 teachers and included the same groups as Cohort 2. Two teachers were typically observed for each school and two whole lessons were observed at each time point where possible. However, these teachers were unable to participate in the intervention during 2020 due to the interruption to schooling caused by COVID-19. As a result, data from this cohort are used for the cross-sectional analysis required to answer RQ1 only. Baseline data from Cohorts 1 and 2 are also used for RQ1 and longitudinal data from these two cohorts are used to address RQ2.

## Research Question 1: The relationship between ICSEA and teaching quality

### Sample

To answer RQ1, we examined the cross-sectional observations collected before any intervention took place (baseline data only). The total cross-sectional sample is displayed in Table 2. As illustrated, a total of 173 NSW government primary schools participated across the cohorts; 13 schools for Cohort 1, 120 schools for Cohort 2, and 60 schools for Cohort 3. The average ICSEA of schools was slightly below 1000 in all cohorts, except Cohort 3, as is characteristic of the Australian government schooling sector (Bonnor & Shepherd, 2017). A total of 832 lesson observations were conducted with the 432 teachers involved in the three cohorts.

**Table 2:** Cross-sectional sample

	Cohort 1	Cohort 2	Cohort 3	Whole Sample
Schools (*n*)	13	120	60	193
ICSEA (Mean, SD)	997, 91	997, 83	1008, 78	1000, 84
Rural (%)	23	38	43	30
Teachers (*n*)	96	221	115	432
Observations (*n*)	190	417	225	832

### Measures

The variables used to conduct the analysis for RQ1 were school ICSEA, a standardised measure of school-level advantage in Australia as described in footnote 1, and QT score. For the analysis, the ICSEA variable was categorised into three groups (ICSEA less than 950, ICSEA 950–1049, and ICSEA above 1049) to gain a nuanced understanding of trends across the ICSEA spectrum. To generate each ICSEA group, we used the national standard deviation for ICSEA, with the cut-off points for each ICSEA group assigned as half a standard deviation either side of the national mean.

QT score was obtained by observing and coding whole lessons using the QT Model. The mean of the 18 elements was used for analysis (range 1–5). 173 lessons were double coded (~ 21% of total observations) and the intraclass correlation coefficient (ICC_(1)-one-way random effects_) was calculated to investigate inter-rater reliability among a large a pool of raters. The ICC for a single measure (single-rater score used for analysis) was 0.808 (95% CI 0.749–0.854), indicating good reliability at the lesson level. The two observations of the same teacher at each time point (which account for 810 of the 832 observations) were investigated for consistency at the teacher level using an intraclass correlation coefficient (ICC_(3)-two-way mixed effects_). The ICC (average measures) for the two observations displayed moderate reliability at 0.602 (95% CI 0.517–0.673), indicating some variability between the two lessons at the teacher level. The raw change in mean between repeated observations was − 0.03 (95% CI: − 0.17–0.10), equating to a negligible difference of − 0.3% (95% CI − 1.8 to 1.3%).

### Analysis

Statistical analyses were conducted using R (R Core Team, 2020), with alpha levels set at *p* < 0.05. Cross-sectional analysis involved fitting a linear regression model using ICSEA as a continuous independent variable and QT score as the dependent variable. To further investigate the relationship between QT score and ICSEA, an additional linear regression was fitted using ICSEA categories. To account for the hierarchical nature of the data, random intercepts were included for school in all models. To ensure the correct *p* value when comparing the three groups, pairwise comparisons (Dunnett contrasts) were used to assess the differences between high ICSEA (> 1049) and low ICSEA (< 950) categories in relation to the reference category of moderate ICSEA (950–1049).

### Results

**Fig. 1:**
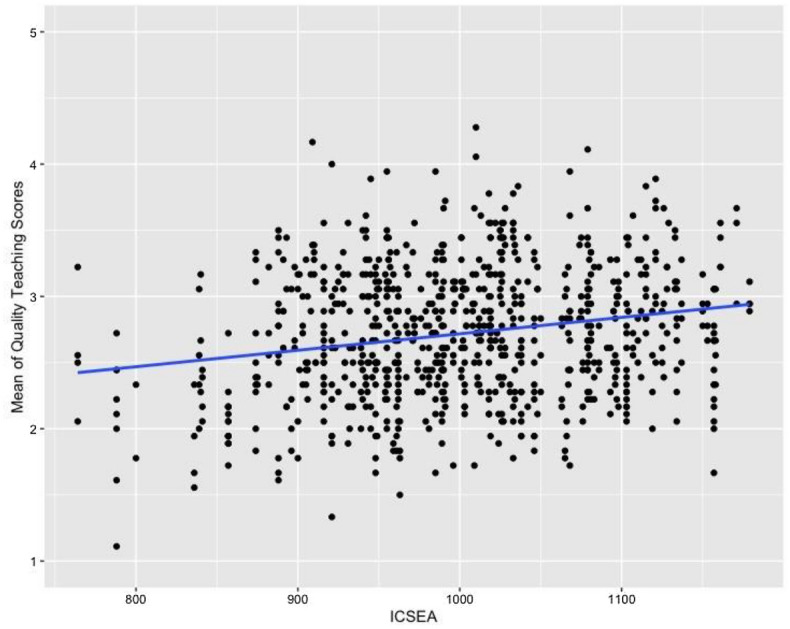
Lesson QT score by ICSEA

Figure 1 displays the scatter plot for lesson QT score by ICSEA and the associated regression line. The relationship between QT score and ICSEA was significant *F*(1, 177) = 22.24, *p* < 0.001, with ICSEA explaining just 3.6% of the variance in QT score at the school level. The Beta coefficient was 0.0012 indicating that the relationship, while significant, was small, with the average QT score increasing by 0.0012, on a 5-point scale, for every 1-point increase in ICSEA.

**Fig. 2:**
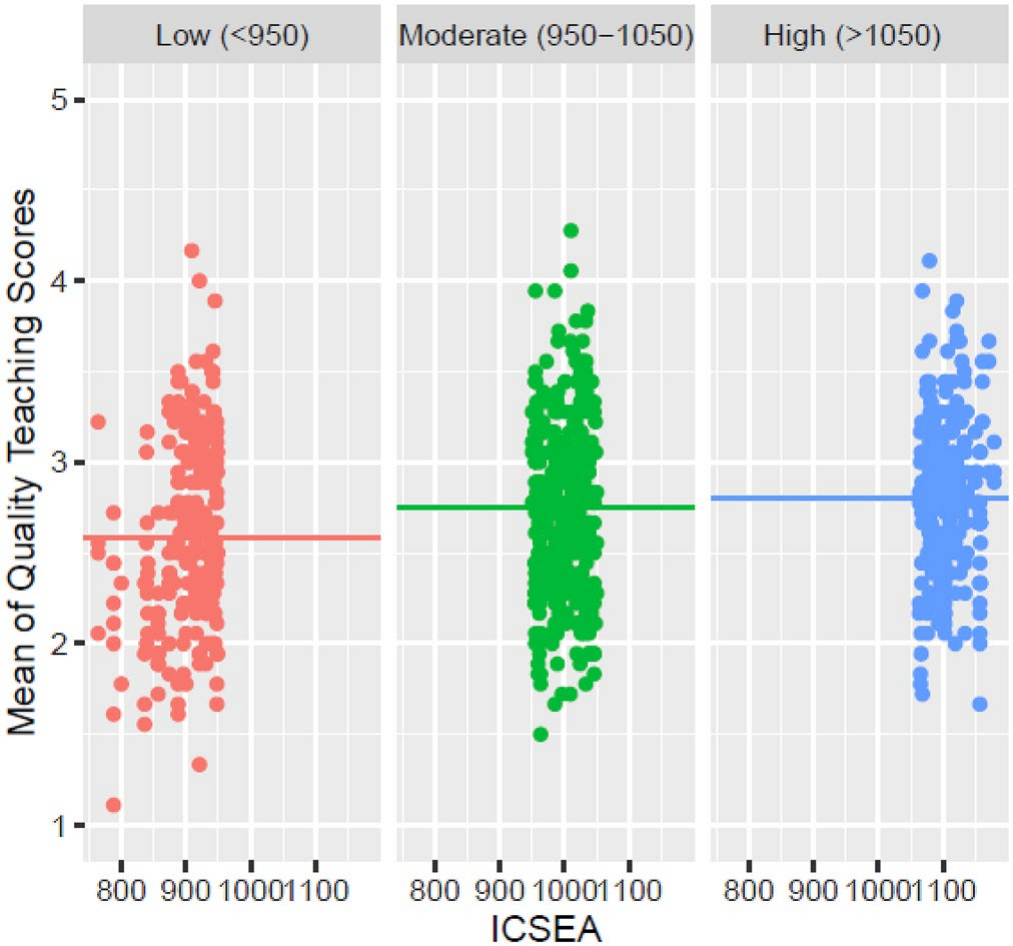
ICSEA category mean QT score

Figure 2 displays the mean QT score for each ICSEA category. Treating the moderate category (ICSEA 950–1049) as the reference category, the linear regression model demonstrated significant differences between the low- and mid-ICSEA categories (mean diff =  − 0.13, *t* =  − 2.346, *p* = 0.040) but no significant difference between the mid- and high-ICSEA categories (mean diff = 0.07, *t* = 1.271, *p* = 0.369). This demonstrates a plateauing effect on the relationship between ICSEA and QT score, with the significant relationship between ICSEA and QT score in the first analysis leveraged by the lowest ICSEA category.

## Research Question 2: Change in QT across the ICSEA spectrum following participation in QTR

### Sample

To answer RQ2, we drew on the Cohort 1 and 2 longitudinal data for all teachers who were randomly assigned to a QTR group. This dataset includes all participants from Cohorts 1 and 2 who undertook QTR. The samples at baseline and follow-up are detailed in Table 3 using the ICSEA groups derived for RQ1. As illustrated in Table 3, the sample contains a total of 69 schools and 174 teachers. A total of 331 observations were conducted at baseline and 289 were conducted after the teachers participated in QTR. Only 3.7% (23/620) of observations were single occurrences (teacher not observed twice).

**Table 3:** Intervention sample

	ICSEA < 950	ICSEA 950–1050	ICSEA > 1050	Whole sample
Schools	19	33	17	69
ICSEA (Mean, SD)	903, 27	997, 30	1118, 33	998, 82
Rural (%)	47	38	3	59
Teachers	40	90	44	174
Observations				
Baseline (*n*)	75	171	85	331
Follow-up (*n*)	59	156	74	289
Total (*n*)	134	327	159	620
Single only	8	10	5	23

### Measures

The variables used to conduct the analysis for RQ2 were ICSEA category (as defined for RQ1) and QT score (pre- and post-intervention).

### Analysis

Statistical analyses for RQ2 were conducted using R (R Core Team, 2020), with alpha levels set at *p* < 0.05. ICSEA category was treated as the independent variable and the mean QT score (for each lesson) the dependent variable. Longitudinal analysis involved fitting a linear mixed model using ICSEA categories, time (pre- and post-intervention), and its interaction term (ICSEA category by time). A repeated statement was included to model the within-subject correlated errors across time (using an unstructured covariance matrix), and random intercepts were included for school to account for the hierarchical nature of the data. Differences in means and 95% confidence intervals (CIs) were determined using the linear mixed models. To ensure the correct *p* value when comparing the three groups across time, pairwise comparisons of the interaction term (Tukey adjustment) were used to assess the differences between high ICSEA (> 1049) and low ICSEA (< 950) categories in relation to the reference category of moderate ICSEA (950–1049).

### Results

Table 4 illustrates the results of the ICSEA category-by-time analysis. As demonstrated, all categories significantly (*p* < 0.05) increased their QT score post QTR. The low ICSEA category increased its average QT score by 0.199 points, the moderate category by 0.205, and the high category by 0.269 (on a 5-point scale). Notably, the group-by-time interaction was not significant (*p* = 0.766). This indicates both that there was positive change in teaching quality across the ICSEA spectrum and the amount of growth was consistent across the spectrum.

**Table 4:** Quality of teaching; ICSEA group-by-time comparison

ICSEA Group	Baseline, mean (95% CI)	Mean change from baseline (95% CI)	Adjusted mean difference (95% CI)^a^	Group × time p	*P* (*pairwise*)
< 950	2.63 (2.48, 2.78)	0.199* (0.029, 0.368)	− 0.006 (− 0.205, 0.192)	0.766	0.950
950–1050	2.76 (2.66, 2.87)	0.205* (0.102, 0.309)	Reference		Reference
> 1050	2.71 (2.56, 2.86)	0.269* (0.114, 0.425)	0.064 (− 0.123, 0.251)		0.498

*Significant at *p* < 0.05

^a^Between-group difference of change score (group change minus reference group change)

## Discussion

In a context where teaching is often positioned as being in ‘crisis’, especially in disadvantaged schools, and with little empirical evidence to justify such claims (Dinham, 2015), this paper provides a long overdue investigation into the relationship between teaching quality and school-level advantage. Using a standardised measure of educational disadvantage (ICSEA), a validated model for measuring the quality of teaching (the QT Model), and whole lessons as the unit of measurement, we investigated whether students in disadvantaged NSW primary schools really do receive poorer quality teaching, as popular political and economic commentators espouse. Next, we examined the relationship between the growth in teaching quality and ICSEA grouping, following the QTR PD intervention. By assessing teachers’ capacity to improve teaching across all ICSEA contexts, we tested the assumption that poorer teachers occupy poorer schools.

At baseline, we found a small but statistically significant relationship between teaching quality and ICSEA, with the average QT score increasing by 0.0012, on a five-point scale, for each one-point increase in school ICSEA. However, the variability in teaching across the ICSEA spectrum was considerable with ICSEA explaining only 3.6% of the variability in QT score. The variability within teachers (ICC = 0.602) contributes, in part, to this variation.

The relationship between ICSEA and QT scores appears to be leveraged downward by the lowest ICSEA group, with no significant difference in average teaching quality score between the two ICSEA groups above 950. This result might be bluntly interpreted as evidence that the most disadvantaged schools have poorer quality teachers. However, our post-intervention analysis counteracts such an interpretation by demonstrating that school ICSEA is not related to improvements in QT score for teachers in these different contexts. That is, the intervention improved teaching quality equally across ICSEA groups, including for those teachers in schools with an ICSEA below 950. Considering that the QTR intervention operates entirely *within* schools, with no external facilitation or oversight required beyond the initial two days of training, this post-intervention result represents phenomenal work being undertaken by teachers across NSW. It not only demonstrates that teachers have capacity for quality teaching in all contexts, but also that the baseline relationship between ICSEA and teaching quality is likely to be a function of school-level, rather than teacher-level, factors.

Furthermore, given the additional challenges teachers face in disadvantaged schools, we contend that the relationship between teaching quality and school ICSEA at baseline is not as large as might be expected. It is well established that teachers in these schools deal with incredibly diverse manifestations of disadvantage everyday (Gorur, 2013). The consequences of such disadvantage, including comparatively under-resourced classrooms, racial inequalities, barriers in access to technology, low parental education levels, and real student poverty (Bonnor & Shepherd, 2017; OECD 2018), are not fully captured in measures like ICSEA (Gorur, 2013). Indeed, as Ravitch (2011) poignantly argues:

Most schools identified as low-performing are sure to enrol large numbers of poor, limited-English, homeless, or transient students. By words and actions, the administration seems to assume that the school gets low scores because it has a bad principal or bad teachers. But the staff may be heroic in the face of daily challenges. (p. 8)

Following Milanowski (2017), we suggest scoring highly on observation frameworks in disadvantaged contexts may simply be more difficult due to the challenges inherent in these contexts. This is not to say that teachers in disadvantaged schools cannot deliver quality teaching, but that the contexts add additional challenges to doing so. The small difference in the quality of teaching between advantaged and disadvantaged schools in this study, we contend, is less a reflection of poorer teachers in poorer schools than a testament to the commitment and hard work of teachers in these often challenging environments.

We argue that our findings are not surprising. Despite relentless political and media positioning of teachers as a major problem driving disparate student achievement, countless academics before us have argued that schools are not failing (Dinham, 2015; Scholes et al., 2017; Thrupp, 2007). For example, there is clear evidence that government schools, in general, perform well on standardised tests once student-background characteristics are accounted for (Sakellariou, 2017)—a significant result given that student-background factors have the largest known influence on student success (Borman & Kimball, 2005; Downey & Condron, 2016; Huang, 2015; Thrupp, 2007). As Downey and Condron (2016) argue:

socioeconomic achievement gaps form primarily *before* formal schooling and schools probably do more to reduce than increase them. If the public and policymakers knew this, they likely would acknowledge that achievement gaps are generated and maintained primarily by forces outside a school’s purview, and they might be more inclined to address the broader social conditions generating those gaps. (p. 217, emphasis added)

Current policies in Australia, as in countries such as the US and the UK, largely focus on accountability, standardisation, school marketisation, and individualisation (Gorur, 2013; Vickers, 2015). Arguably, these policies have done little to address underlying inequalities, instead exacerbating them, as is evident in school socioeconomic segregation (Bonnor & Shepherd, 2017). Meanwhile, the salaries we pay teachers have fallen relative to other occupations (Leigh & Ryan, 2008), while teacher workload, stress, criticism, and turnover have increased considerably (Garrick et al., 2017). Policies that portray schools and teachers as failing do little more than serve as a distraction for real sources of difference (Downey & Condron, 2016).

Our finding that teachers in all contexts improved the quality of teaching they delivered signals the value of powerful professional development, like QTR, in all contexts. We acknowledge the potential role reactivity may have played in these results—teachers engaged with a model of pedagogy that was used to measure the quality of their teaching subsequently performed better after participation in QTR. However, the fact that participation has now been linked with increased student achievement (Gore et al., 2021) mitigates this potential limitation. Additionally, the model does not prescribe a set of procedures or routines for teachers to follow; rather, it provides a conceptual and practical basis for teachers to analyse and refine their own practice, regardless or topic, subject area, or student grade level.

A few other limitations should be noted. First, while inter-rater reliability for the QT score is considered ‘good’ (ICC = 0.808), the ‘moderate’ reliability at the teacher level (ICC = 0.602) indicates variability between lessons taught by the same teacher (i.e. 60% of teaching quality, as measured by the QT Model, is captured using two observations). Increasing the number of observations might reduce variability in QT score and increase the explanatory power of the models presented, but without such data appropriate caution should be used in generalising these results. Second, given that our results pertain only to the NSW government school sector, further research could usefully undertake cross-sector, cross-state, and even international comparisons. Given differences in school funding arrangements, teacher accreditation, and regulation of teacher professional development, generalisation of our findings to different locations cannot be made without further investigation. Third, identifying little difference in the quality of teaching across contexts does not mitigate the need for improving teaching in general. Indeed, given that the average QT score is 2.7 on the 1-to-5 scale, our data highlight a potential to improve teaching across the board, not just in disadvantaged schools. A similar conclusion regarding this need was also reported by Graham et al. (2020) in their recent study of teacher experience. However, this is not to say that teachers can just be expected to improve without being given adequate conditions to do so. Lastly, as only a select number of teachers from each school participated in QTR, the results may reflect a more motivated cohort of teachers unrepresentative of whole schools. Anecdotally, however, a sizeable number of participants spoke of being ‘volunteered’ by their principal and/or being initially reserved about their participation (e.g. Gore & Rickards, 2020) indicating that many of the teachers in the study were not particularly motivated to be involved.

Additional research in this area could investigate how improvements in teaching quality meditate improvements in student outcomes. Results from our most recent randomised controlled trial demonstrated that participating in QTR produced significant improvement in student mathematics outcomes, with a stronger effect in disadvantaged schools (Gore et al., 2021). More research is warranted on *how* improvements in teaching translate into increased student learning. Nevertheless, the policy implication is clear: investing in meaningful teacher professional development can make a difference for students in disadvantaged schools.

## Conclusion

The socio-educational achievement ‘gap’ is a longstanding global problem. Expecting individual students to overcome systemic handicaps (Huang, 2015), teachers to ‘save the poor’ (Berliner, 2013; Borman & Kimball, 2005), and the ‘school marketplace’ to fix educational ills (Gorur, 2013) are fraught policies that do not address the fact that Australia is becoming a more unequal, more highly segregated society (Bonnor & Shepherd, 2017; Perry, 2018). Indeed, many recent policies have only exacerbated existing inequalities, resulting in compounding levels of disadvantage (Bonnor & Shepherd, 2017) that teachers are expected to alleviate. Our results show that teachers, in general, are doing well and that teachers who work in the most challenging schooling environments are no exception.

At the same time, as indicated by the general improvement in quality of teaching following participation in QTR, we contend that sector-wide improvement is possible for all. Policymakers here and internationally should note that, in our study, improvement was driven by respect for the profession and creating opportunities for teachers to undertake powerful PD—not by regulation, standardisation, or top-down accountability.

Finally, in the light of our findings, we argue that broader policies aimed at alleviating disadvantage and levelling the education playing field are sorely needed to reduce socio-educational inequalities. The conditions facing students and their families in many disadvantaged contexts, as well as teachers’ work lives in such contexts, require holistic and systemic attention. For too long, the poor have been blamed for circumstances not of their making and governments have failed to fully implement needs-based school funding structures (Bonnor & Shepherd, 2017). Education can make a difference, but we must support teachers in their endeavours to do so.

## Footnotes

1 ICSEA is a standardised scale used for measuring school-level advantage in Australia. It includes parent education and occupation, proportion of Indigenous students and school geographic location. It has a mean of 1000 and standard deviation of 100. However, the mean ICSEA of government schools is below 1000 (Bonnor & Shepherd, 2017) demonstrating concentrated levels of disadvantage in the government schooling system.
